# Scientific approach and attitudes among clinically working physiotherapists in Sweden -a cross sectional survey

**DOI:** 10.1186/s40945-023-00173-6

**Published:** 2023-10-09

**Authors:** Frida Eek, Pernilla Åsenlöf, Kjerstin Stigmar

**Affiliations:** 1 Department of Health Sciences, Lund University, Lund, Sweden; 2 Department of Women’s and Children’s Health, Physiotherapy and Behavioural Medicine, Uppsala University, Uppsala, Sweden

**Keywords:** Physiotherapy, Physical therapy, Evidence based practice, Science

## Abstract

**Background:**

Evidence based medicine (EBM) should be an endeavor within all healthcare professions. Knowledge and understanding of science are important prerequisites of EBM.

**Objective:**

The aim was to examine and compare perspectives on science and perceived inhibiting and facilitating factors for the assimilation and implementation of scientific information among clinically working specialist- and non-specialist physiotherapists in Sweden.

**Methods:**

A cross-sectional survey study was conducted via a web-based questionnaire. Clinically active physiotherapists in Sweden were invited to participate. Attitudes and perspectives were compared between physiotherapists with completed or on-going specialist training, and non-specialists.

**Results:**

In total, 1165 physiotherapists responded to the survey (75.5%, (*n* = 870) women, mean age 44.8 (SD 12.1), whereof 25.5% (*n* = 319) with completed or ongoing specialist training). The majority of participants had a high interest in science but did not consider a general scientific approach to be applied within physiotherapy. The main perceived inhibiting factor for a clinical practice more based on scientific evidence was lack of time. Specialists had in general higher interest and ability to interpret and evaluate science, and prioritized scientific evidence to a higher extent.

**Conclusion:**

Among respondents, a scientific approach was considered valuable within physiotherapy but not considered fully applied in practice. The higher interest and perceived ability to interpret science among specialists indicates that further education and specialist training can increase both interest and understanding of science among physiotherapists.


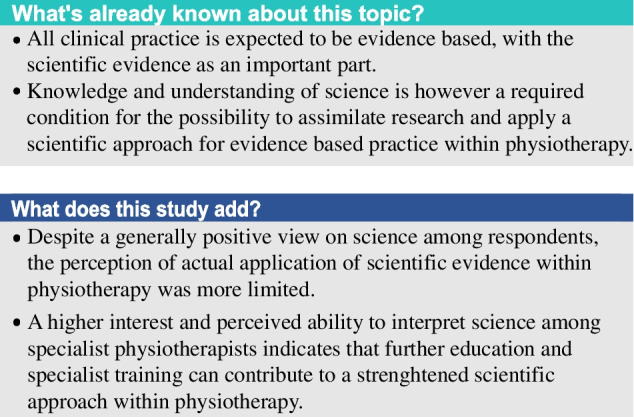


## Introduction

Evidence based medicine (EBM) is and should be an endeavor within all healthcare professions. In 1996, Sackett et al. defined EBM as "conscientious, explicit, and judicious use of current best evidence, combined with individual clinical expertise and patient preferences and values, in making decisions about the care of individual patients" [1]. Evidence based medicine thus rests on three legs; scientific evidence, proven clinical experience and expertise, and patient preferences. Additional aspects such as benefit weighed against risk, resource availability, practical feasibility, etc. should also be weighed into clinical decision making [2]. Even if all aspects must be weighed in evidence-based care decisions, the scientific evidence base weighs heavily in the context, in cases where it exists.

In the 1970s and 1980s, the need for strengthening the empirical practice of medicine was highlighted. In the early 1990s, the term EBM was initially introduced, and started as a movement [2, 3]. Initially, the focus was on educating clinicians in the understanding of scientific results, increasing knowledge about and the use of published scientific studies in clinical practice. The development then came to also include evaluation of studies and results, which further increased the need for knowledge and understanding of the scientific methodology. This progression has continued, and contribute to strengthening both the quality of, and the application of EBM in the clinic [2].

There are however indications that physiotherapists often do not fully apply the EBM process, and prefer to obtain knowledge from colleagues or social networks rather than from the scientific literature [4]. In a qualitative study of Swedish physiotherapists, the participants mentioned that knowledge of scientific methods, including the ability of critical/analytical review, improves the conditions for applying research results in the clinic [5]. Internal motivation for application of EBM is related to genuine curiosity and willingness to learn from research, and the that research use enables provision of best possible care for the patient [6]. A quantitative study of Swedish physiotherapists' attitude, knowledge and approach to EBM showed that approximately 90% agreed that EBM is necessary in clinical practice, and 83% agreed that scientific evidence is helpful in decision-making [7]. At the same time, 90% agreed that they wanted to learn or improve the skills required to fully apply EBM in the clinic. Of the participating physiotherapists, 44% read fewer than two scientific articles per month. In another Swedish study, 44% of the participants had read scientific articles only a few times during the current year [8]. Commonly stated barriers to practicing EBM were lack of time, lack of advisers, lack of knowledge and lack of interest from supervisors. This is also confirmed in two systematic reviews where the most often indicated obstacles to the application of EBM were found to be lack of time, inability to understand statistics, misperceptions of EBM, lack of support from the employer and lack of both resources and interest [9, 10].

In other international studies, physiotherapists have indicated insufficient teaching and knowledge in scientific methodology as one of the main barriers to practicing EBM [11–14]. In a Brazilian study, physiotherapists considered the lack of access to full-text articles to be the biggest barrier to implementing EBM, but even there a large proportion indicated insufficient knowledge and understanding, and lack of research experience as barriers to implementing EBM [14]. Also, in studies from Colombia and Austria, insufficient knowledge and understanding of research and statistics were indicated as main barriers to the practice of EBM among physical therapists [12, 13].

In Sweden, physiotherapy education is a three-year bachelor program [15]. After completed education, the formal registration is done by the National Board of Health and Welfare, which is the authority that authorize all registered professions in health care, such as physicians, midwifes, psychologist etc. Master programs in physiotherapy are available at some Universities. Physiotherapist can also obtain clinical specialization in a wide range of areas. The specialization encompasses a two–three-year supervised training in clinical practice as well as completed studies on advanced level (at least a one-year Master) within the area of specialization and is approved by The Swedish Association of Physiotherapists [15]. In Sweden, there were 18 541 registered physiotherapists in working age 2018, whereof around 80% are employed in health care [16]. Currently, approximately 6% of them are specialists [17]. There is no need for referrals to physiotherapy treatment in Sweden and in primary health care patients with musculoskeletal disorders are directed through open access to physiotherapy, as first line treatment. This emphasizes the importance of qualification for EBM in physiotherapy care.

Since science is an important prerequisite of EBM, it is valuable to obtain more knowledge of physiotherapists´ current attitudes, and ability to use and consume research. The current study aimed to examine perspectives on science related to clinical practice, as well as perceived inhibiting and facilitating factors for the assimilation and implementation of scientific information among clinically working physiotherapists in Sweden. A further aim was to compare the perspectives between physiotherapists with and without specialist training.

## Materials and methods

### Design and population

A cross-sectional study was conducted via a digital/web-based survey. The study was directed to clinically active and registered physiotherapists in Sweden.

### Sample selection and data collection

An invitation to participate in the study was sent out to all members (approx. 12,500) within the professional association and trade union The Swedish Association of Physiotherapists (“Fysioterapeuterna”), including all clinical employment areas within physiotherapy. The survey was sent out by The Swedish Association of Physiotherapists in May 2022 and was open until September 2022. A reminder was sent out in August. Also, subgroups in specific areas of practice within The Swedish Association of Physiotherapists sent out invitations to their members. The invitation included a separate link and encouragement to spread the link/invitation to colleagues who were not members in the professional association/trade union. The link and invitation to the survey was also spread via different channels in social media (Facebook, twitter) with encouragement to spread the invitation to colleagues, to also reach physiotherapists who were not members of the professional association/trade union.

### Questionnaire

The digital survey was constructed in Sunet Survey. The content was constructed in collaboration between the three authors and was also pilot tested by 13 physiotherapists in clinical practice to receive feedback. Some minor changes were made according to their feedback. The survey included questions within three topic fields, as presented below, with attention on perspectives and attitudes towards science related to clinical practice. The survey also included demographic/background questions regarding sex, age, clinical experience, employment, education level (Bachelor, Master, or PhD) and specialist training (completed or ongoing).

#### Perspectives on implication of science in clinical practice

The survey included questions about how interested the participant was in science; if/how their interest had changed during clinical practice; how important they consider a scientific approach is for quality of clinical interventions, and to what degree they consider their own, as well as the general clinical practice within physiotherapy in Sweden, is based on scientific evidence. The response alternatives were five categorical Likert scales. An open-ended question was asked regarding the reason for change in interest of research during clinical practice. Further, agreements for main facilitating and inhibiting factors for clinical implications were stated. Participants were also asked to rank the priority of basis for clinical practice; current science, treatment protocols, own clinical experience, knowledge from education/courses, and patients’ preferences.

#### Uptake and perceived abilities for assimilation of science

Participants were asked how often they read scientific articles about health, medicine, or physiotherapy and which suggested reasons they agreed with for reading, or not reading, scientific papers. They were also asked about their perceived ability to understand the structure and performance of scientific studies, to evaluate the methodology/performance, and to interpret statistical results, with response alternatives of a five categorical Likert scale. Finally, they were asked if they consider their knowledge/understanding of research sufficient to make decisions regarding treatment based on a scientific basis, and if they considered that they received sufficient education in scientific methodology during their undergraduate program.

### Data management, statistics, and quantitative content analysis

Specialist physiotherapists were defined as having completed or ongoing specialization within physiotherapy. Most five categorical Likert scales were dichotomized (into e g “quite/very” and “moderately/little/not at all” interested) and presented as percent (%) and number (n) for each category in the total sample, and among specialists vs non-specialists. Comparisons between specialists and non-specialists of proportions within dichotomized/categorical variables were performed with chi-square test. The perceived ability to understand and evaluate aspects of science was also presented with mean score of the scale from 1 (very low) to 5 (very high) and compared with independent samples t-test. Number of read papers/month was presented with both mean and median, but due to the skewed distribution the comparison was done with Mann–Whitney U test. The rank order of each of the five foundations for clinical practice was defined from 1 (lowest priority) to 5 (highest priority), and the mean rank for each foundation was presented and compared between specialists and non-specialists with independent samples t-test. Also, the proportion of “highest rank” selection for each foundation was presented and compared between specialists and non-specialists with chi square test. Since specialists in the sample had a longer clinical experience, complementary analyses were performed including adjustment for duration of clinical experience (ANOVA for scale outcomes and logistic regressions for binary outcomes). However, since significant differences remained with no apparent change in estimates, the basic/initial non-adjusted analyses were applied and presented. Also stratified analyses of responders via email invitation from The Swedish Association of Physiotherapists vs social media was performed, but no clear differences were observed (results not presented).

For open-ended question regarding perceived reason for increased or decreased interest in science, the comments were read by two authors and categorized into sub- and main categories. For comments including different perspectives, each perspective was separately categorized. The number of comments within each category were presented.

### Ethics

All data were collected anonymously. Since no intervention was performed on a research person and no personal data/information was handled, the study is not covered by the ethics review law. An application was however sent to the Swedish Ethical Review Authority (Dnr 2022-00815-01), which confirmed the study to not be covered under the ethical law (Ethics review act) and instead provided an advisory opinion with no contradictions to the performance of the study.

## Results

In total, 1165 physiotherapists responded to the survey. Among participants, 76.6% (*n* = 892) responded to the invitation via The Swedish Association of Physiotherapists, which corresponds to a response rate of approximately 7% of the members. Remaining 23.4% (*n* = 273) of participants answered the survey via social media invitation. The majority of all respondents (75.5%, *n* = 870) were women, and mean age was 44.8 (SD 12.1). Among all participants, 25.5% (*n* = 319) had a completed or ongoing specialist training within physiotherapy. The mean age was higher among specialists than non-specialists, and the specialists also had longer clinical experience (Table 1).

**Table 1:** Sample description

	Total *n* = 1165	Not specialists *n* = 840	Specialist^a^ *n* = 319
**Sex % (n) Total *n* = 1153**			
Female	75.5 (870)	75.1 (626)	76.8 (241)
Male	23.6 (272)	24.0 (200)	22.6 (71)
Don’t want to declare	1.0 (11)	1.0 (8)	0.6 (2)
**Age (total *n* = 1159)** Mean (sd) years	44.8 (12.1)	43.1 (12.0)	49.1 (11.0)
**Employer/employment total n 1164**			
Private	20.6 (240)	19.3 (162)	23.8 (76)
Municipality	12.4 (144)	16.5 (139)	1.6 (5)
Region -university hospital	15.1 (176)	12.3 (103)	22.6 (72)
Region -other operation	36.7 (427)	40.7 (342)	26.6 (85)
Self employed	13.2 (154)	9.2 (77)	23.5 (75)
Public	0.9 (11)	1.0 (8)	0.9 (3)
Other	1.0 (12)	1.1 (9)	0.9 (3)
**Highest academic exam % (n) total *n* = 1159**			
Basic (previous) PT (2–2.5 years)	6.4 (74)	8.2 (69)	0.9 (3)
Bachelor degree BSc (3 years)	55.2 (640)	72.6 (608)	9.7 (31)
MSc one year (Magister)	20.5 (238)	11.4 (95)	45.0 (143)
MSc (two year)	11.9 (138)	5.6 (47)	28.6 (91)
PhD	6.0 (69)	2.2 (18)	15.7 (50)
**Years since exam total *n* = 1159**			
Mean (sd)	18.4 (11.7)	16.7 (11.5)	22.8 (11.0)
Md (Q1-Q3)	17 (8–28)	15 (7–26)	22 (14–32)
**Years of clinical experience total *n* = 1154**			
Mean (sd)	17.6 (11.4)	15.9 (11.2)	22 (10.7)
Md (Q1-Q3)	16 (8–27)	14 (6–25)	22 (13–31)
**Specialist within PT** % (n) **total *n* = 1159**			
No	72.5 (840)	–	–
Yes	20.2 (234)	–	–
Ongoing specialisation	7.3 (85)	–	–

*PT* Physiotherapy, *BSc* Bachelor of Science, *MSc* Master of Science, *PhD* Doctor of Philosophy (within medicine)

^a^Ongoing or completed specialist training within physiotherapy

### Interest and perspectives on implication of science in clinical practice

The majority of participants were quite or very interested in science and considered their interest to have increased during their clinical practice. Both high and increased interest were more often reported by specialists than among non-specialists (Table 2). The reported reasons for increased interest (open comments) were categorized into four main categories with six, two, five, and two sub-categories respectively (Table 3), and the reasons for decreased interest were categorized into two main categories with four and two sub-categories respectively. The main reported reason for increased interest was a desire to increase and update knowledge of scientific evidence for best clinical practice (*n* = 241), and that the clinical experience had increased interest (*n* = 176). Further reasons for increased interest were own continuous education (courses, specialist training and research practice/PhD) (*n* = 159) and the general development within both science and the profession (*n* = 85). The main reported reasons for decreased interest were lacking time and access to scientific evidence (*n* = 63).

**Table 2:** Perspectives on science in relation to clinical practice among specialists and non-specialist physiotherapists

	Total *n* = 1165	Not specialists *n* = 840	Specialists^*^ *n* = 319	*p*-value^**^
**Interest in science** % (n)				
Quite/very interested	87.2 (1010)	83.7 (703)	96.5 (307)	
Moderate/Little/not interested	12.8 (148)	16.3 (137)	3.5 (11)	< .001
**Change in interest during clinical experience** % (n)				
Increased	66.1 (763)	59.8 (500)	82.7 (263)	
Similar/No change	33.9 (391)	40.2 (336)	17.3 (55)	< .001
**Importance of scientific approach for the quality of clinical interventions %(n)**				
High/very high importance	91.5 (1054)	91.0 (759)	92.8 (295)	
Moderate/low/none	8.5 (98)	9.0(75)	7.2 (23)	0.408
**Perception of to what degree own clinical practice is based on scientific evidence/basis %** (n)				
High/very high degree	69.4 (801)	64.5 (540)	82.3 (261)	
Moderate/low/not at all/don’t know	30.6 (353)	35.5 (297)	17.7 (56)	< .001
**Perception of to what degree clinical practice among Swedish PTs generally is based on scientific evidence/basis %** (n)				
High/very high degree	39.1 (451)	38.6 (323)	40.3 (128)	
Moderate/low/not at all/don’t know	60.9 (703)	61.4 (513)	59.7 (190)	0.637

^*^Specialists = ongoing or completed specialist training within physiotherapy

^**^Comparison between specialists and non-specialists; Chi square test

**Table 3:** Categorised perceived reasons for changed interest in science

Reason for increased interest		
Main categories	Subcategories	Reported comments (n)
Desire to increase and update knowledge of scientific evidence for best clinical practice	Want to develop own knowledge	69
	Keep oneself updated	36
	Get an evidence base/work evidence based	36
	Be able to help patients in the best way	33
	Interest in connecting clinic and science	15
	Have a critical view/to develop the profession	52
Own further education; courses or research	Involvement and interest in performing research	60
	Further education/own specialisation	99
Own experience from the clinic emphasize the needs	Own clinical experience	43
	More experience gives greater insight into the need to learn more	58
	Specific clinical area has attracted interest	17
	Perceived weaknesses within the profession (including previous basic education)	41
	Inspiring colleagues	17
The development of science and the profession	Science within physiotherapy has increased and EBM is more requested	75
	Accessibility to science	19
**Reason for decreased interst**		
Main categories	Subcategories	Reported comments (n)
Lack of time and access to evidence	Lack of time/space within work time	36
	Science does not have (good) answers, or does not exist	20
	Lack of access	< 10
	Difficult to find/interpret/read	< 10
Lack of inspiration	Too much focus on research	< 10
	Disinterested colleagues/employers	< 10

Most participants (91.5%) considered a scientific approach to have high or very high importance for clinical practice, but 60.9% thought that the clinical practice of physiotherapists in Sweden is generally based on a scientific basis to a moderate/low degree, or not at all (or didn’t know to what degree). There was no significant difference regarding these opinions between specialists and non-specialists. A significantly higher proportion of specialists did however consider their own clinical practice to be based on scientific evidence (Table 2).

**Fig. 1:**
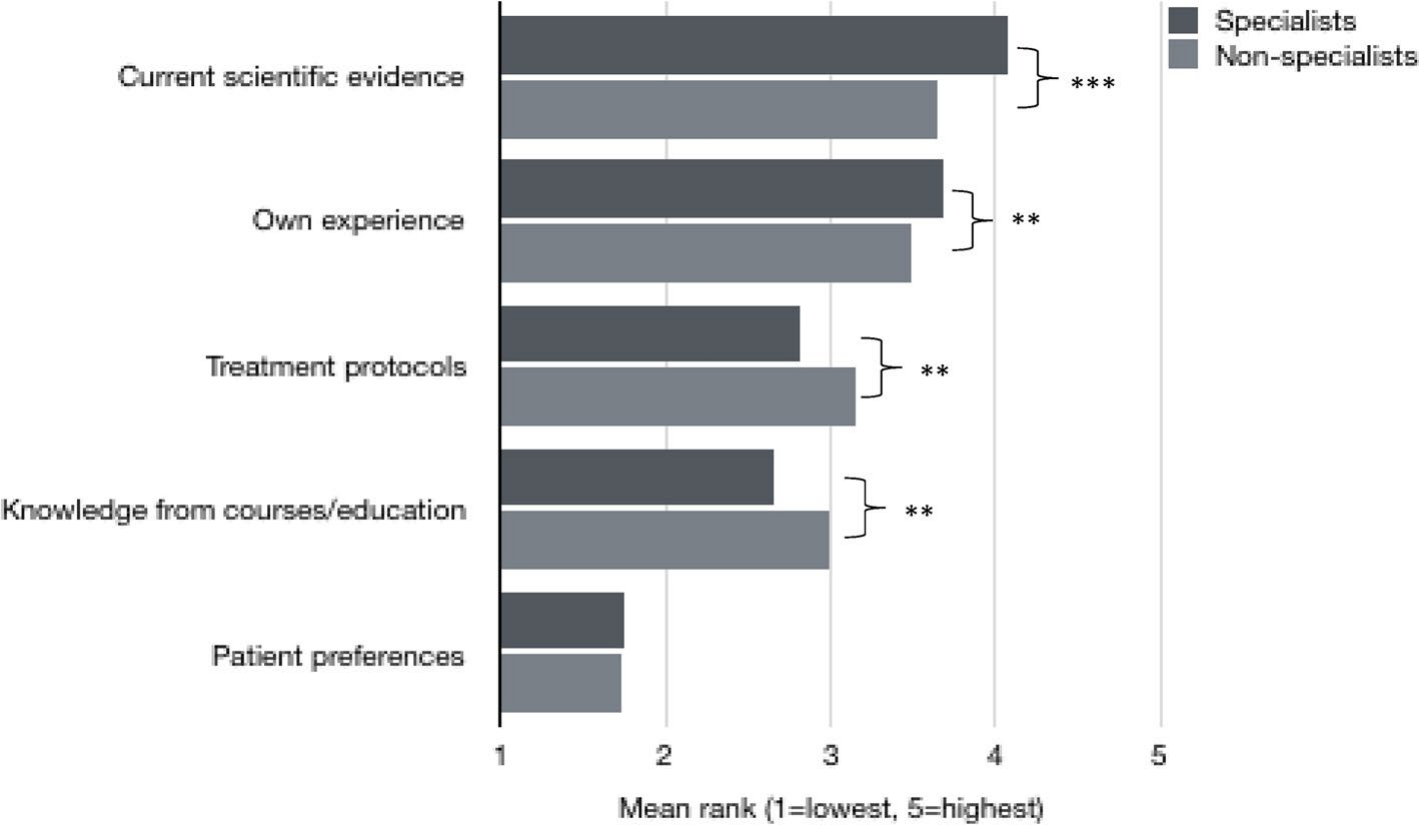
Mean rank order of main/primary fundament for clinical treatment among specialists (*n* = 265) and non-specialist physiotherapists (*n* = 693). 5 = Highest rank/main priority; 1 = Lowest rank/priority. Difference in mean rank order between specialists and non-specialists: *p $\le$ 0.05, **p < 0.01, ***p < 0.001

Among the participants, current scientific evidence was ranked as highest prioritized foundation for selection of clinical treatments (Fig. 1). The second highest ranked priority was own clinical experience followed by existing treatment protocols and knowledge from courses/education. The lowest ranked foundation was patient’s preference. Specialists ranked scientific evidence as higher priority than non-specialists (*p* < 0.001) with a significantly larger proportion choosing it as highest rank/priority (46% vs 32%; *p* < 0.001). Own clinical experience had a higher mean ranked priority among specialists (*p* = 0.005), but similar proportion chose as highest priority (26.4% vs 25.5%, *p* = 0.805). Non-specialists ranked treatment protocols and knowledge from courses/education as higher priority than specialists (*p* <  = 0.003) and larger proportions chose as highest rank/priority (treatment protocol: non-specialists 24.7% vs specialists 17.0%, *p* = 0.012; courses/education 15.2% vs 7.9%, *p* = 0.003). The ranked priority of patient’s preference was approximately similar between the groups (*p* = 0.913) (Fig. 1) with a low proportion choosen this alternative as highest rank/priority in both groups (2.6% vs 2.6%, *p* = 1.000).

### Intake and perceived abilities for assimilation of science

**Fig. 2:**
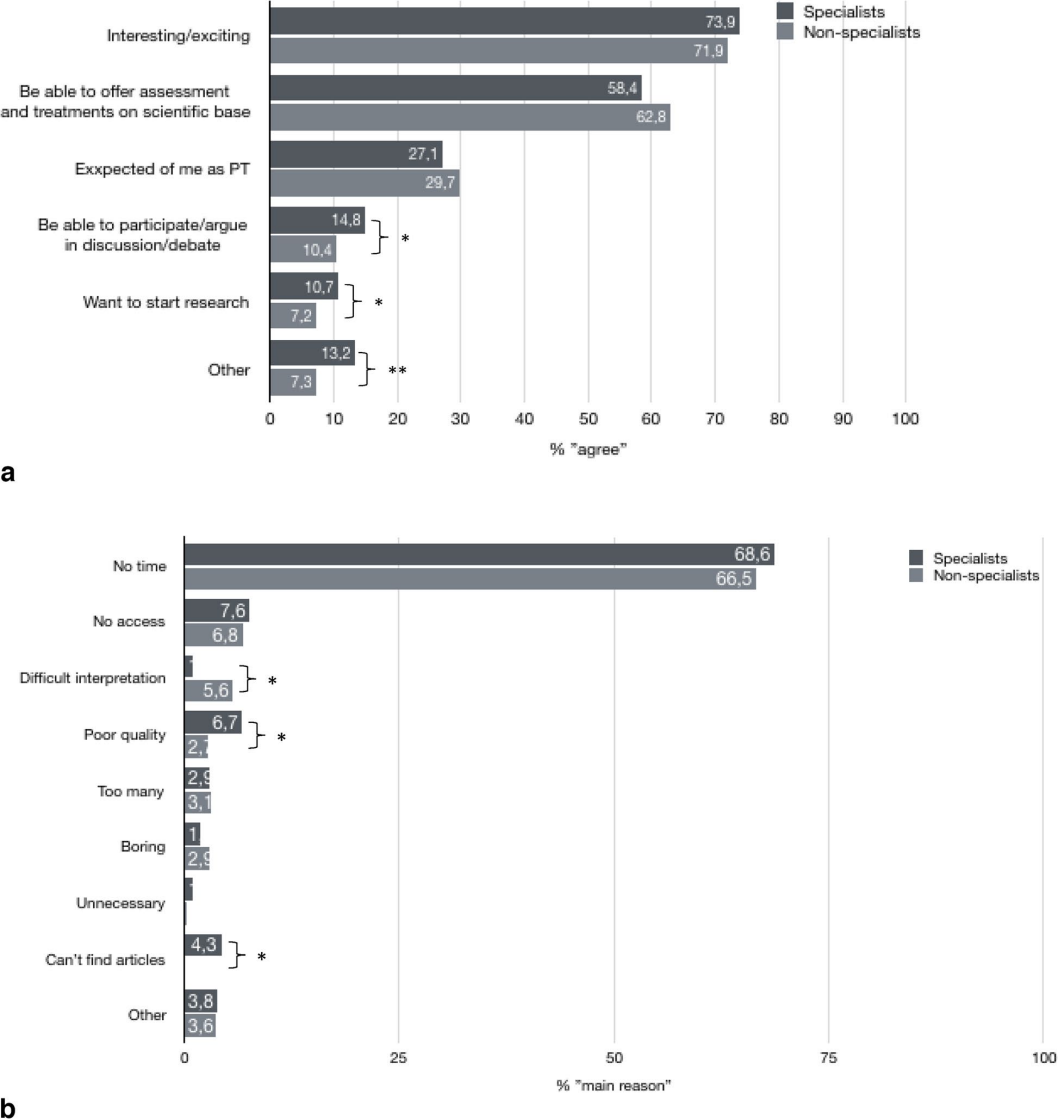
**a** Reasons (agree, multiple answers possible) for reading scientific papers among physiotherapists in clinical practice, specialist (*n* = 317) and non-specialists (*n* = 808). Difference in proportions between specialists and non-specialists: *p $\le$ 0.05, **p < 0.01, ***p < 0.001. **b** Reported (agreed) *main* reason for *not* reading scientific papers more often, among those reading never, rarely or sometimes (non-specialists (*n* = 585) specialists (*n* = 105)). Difference in proportions between specialists and non-specialist physiotherapists: *p $\le$ 0.05, **p < 0.01, ***p < 0.001

The median number of scientific papers read during a regular month was 2 (IQR 1–4) (Table 4). Specialists reported significantly higher number of papers read during a month; Md 3 (IQR 2–5) compared to Md 1(IQR 1–3) among non-specialists (*p* < 0.001). The options most participant agreed to as reasons for reading scientific papers was that it is found interesting or exciting, and to be able to offer assessments and treatments on a scientific basis (Fig. 2a). Among those not reading scientific papers regularly or often, lack of time was considered as the main reason (Fig. 2b).

Among the participants, the perceived ability to understand the structure and performance of studies, such as study design, was rated higher than the ability to evaluate the methodology, such as identify bias etc. (Table 4). Interpretation of statistical results was the lowest rated perceived ability in the sample. Specialists perceived their ability significantly higher than the non-specialists both regarding understanding of structure, evaluation of methodology and interpretation of statistical results (p’s < 0.001). Less than one third (30.2%) of participant thought that they learned enough about scientific methodology during their undergraduate program, while the majority (67.8%) of the sample considered their current knowledge/understanding of research as sufficient enough to make decisions regarding treatment based on a scientific basis, with a higher proportion among specialists (*p* < 0.001).

**Table 4:** Assimilation and perceived ability of interpretation of scientific results among specialist- and non-specialist physiotherapists

	Total *n* = 1165	Not specialist *n* = 840	Specialist^*^ *n* = 319	*p*-value^**^
**Frequency of reading scientific articles about health, medicine, or physiotherapy** % (n)				
Regularly/often	40.5 (469)	30.4 (255)	67.1 (214)	
Sometimes	39.2 (454)	43.8 (368)	27.0 (86)	
Rarely/never	20.4 (236)	25.8 (217)	6.0 (19)	< .001
**Number of scientific articles read during a normal month**				
Mean (SD)	3.6 (6.0)	3.0 (5.9)	5.2 (6.2)	< .001
Md (IQR)	2 (1–4)	1 (1–3)	3 (2–5)	< .001
**Perceived ability to understand the structure and performance of scientific studies (study design, etc.)**				
High/very high % (n)	42.2 (487)	48.5 (406)	82.3 (261)	< .001
Mean (SD) score 1 (very low) -5 (very high)	3.7 (0.8)	3.5 (0.7)	4.1 (0.7)	< .001
**Perceived ability to evaluate the methodology of scientific studies (identify bias, assess quality, etc.)**				
High/very high % (n)	40.6 (468)	31.5 (263)	64.7 (205)	< .001
Mean (SD) score 1 (very low)-5 (very high)	3.4(0.8)	3.2 (0.8)	3.8 (0.7)	< .001
**Perceived ability to interpret statistical results from scientific studies? (significance/*p*-value, effect sizes, risk measures, confidence intervals, etc.)**				
High/very high % (n)	25.6 (295)	18.6 (155)	43.9 (140)	< .001
Mean (SD) score 1 (very low)-5 (very high)	3.0 (0.9)	2.8 (0.9)	3.5 (0.8)	< .001
**Perceived ability to translate/implement results from scientific studies in your clinical practice**				
High/very high % (n)	46.1 (521)	37.7 (307)	67.7 (214)	< .001
Mean (SD) score 1 (very low)-5 (very high)	3.4 (0.8)	3.3 (0.7)	3.8 (0.7)	< .001
**Sufficient knowledge/understanding or research for decision making regarding treatments**				
Yes % (n)	67.8 (785)	61.3 (515)	85.2 (270)	
No	9.1 (105)	11.5 (97)	2.5 (8)	
Don’t know/can’t judge	23.1 (267)	27.1 (228)	12.3 (39)	< .001
**Sufficient education in scientific reading and understanding during undergraduate studies**				
Yes I learned enough	30.3 (349)	31.8 (266)	26.3 (83)	
Yes, it was offered but I didn't take in the knowledge	6.4 (74)	6.3 (53)	6.6 (21)	
Yes I learned but have forgot most of it	23.2 (267)	28.2 (236)	9.8 (31)	
No, the education within this topic was insufficient	32.6 (376)	28.3 (237)	44.0 (139)	
Don’t remember	7.5 (86)	5.3 (44)	13.3 (42)	< .001

^*^Specialist = ongoing or completed specialist training

^**^Comparison between specialists and non-specialists. Chi square test for categorical outcomes; Mann–Whitney U test for non-parametric rank comparison, and independent samples t-test for mean comparison of scale outcomes

**Fig. 3:**
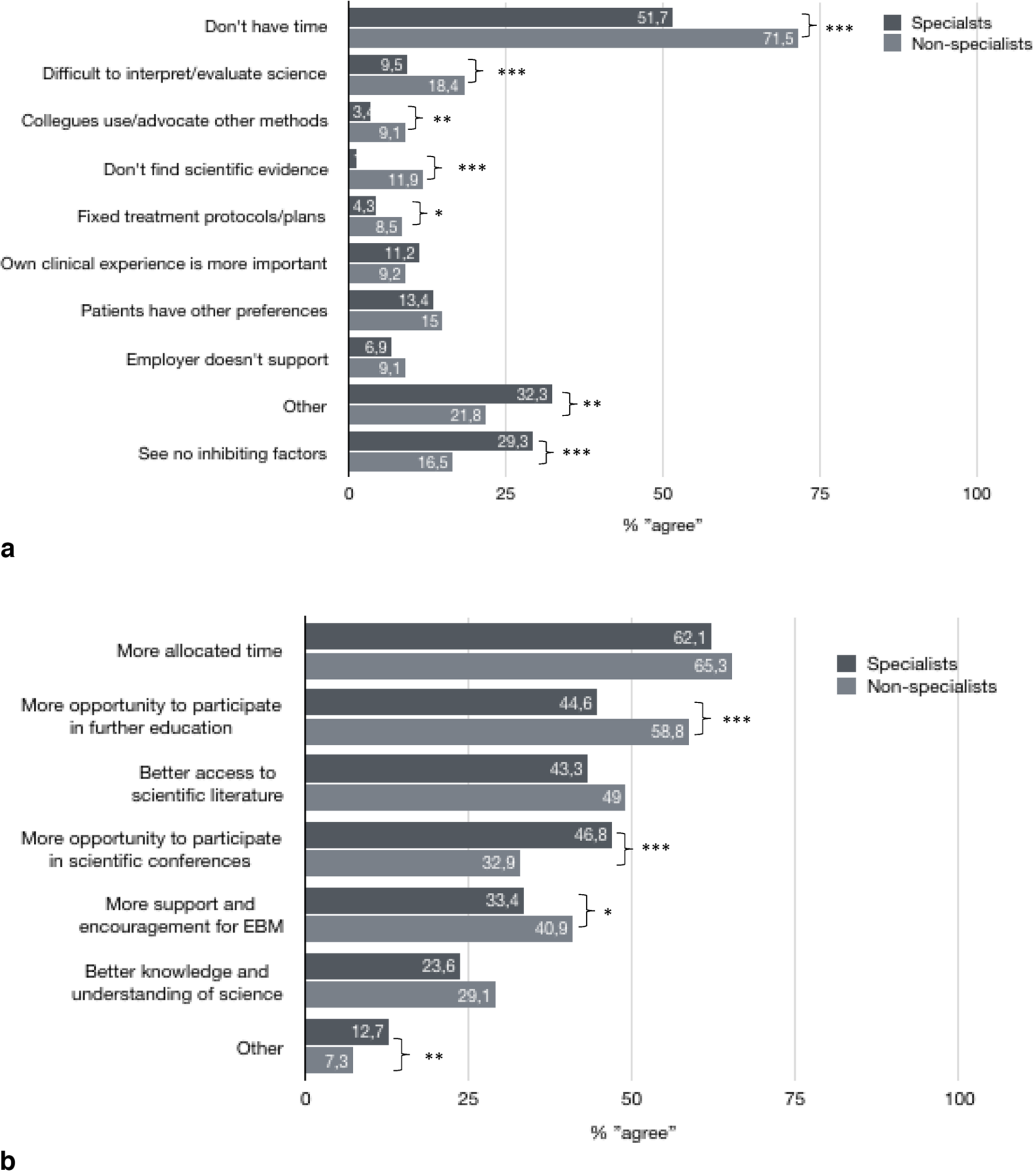
**a** Perceived barriers for clinical practice more based on scientific evidence among non-specialist (*n* = 751) and specialist (*n* = 232) physiotherapists (multiple answers possible). Difference in proportions between specialists and non-specialists: *p $\le$ 0.05, **p < 0.01, ***p < 0.001. **b** Perceived facilitators for a scientific approach among non-specialist (*n* = 838) and specialist (*n* = 314) physiotherapists (multiple answers possible). Difference in proportions between specialists and non-specialists: *p $\le$ 0.05, **p < 0.01, ***p < 0.001

The main perceived inhibiting factor for a clinical practice more based on scientific evidence was lack of time (Fig. 3a). More allocated time, more possibility to participate in further education, and better access to scientific literature was the factors most frequently reported as facilitating conditions for a scientific approach in clinical practice (Fig. 3b).

## Discussion

The majority of the physiotherapists who participated in the survey had a high interest in science. There were however differences between specialized physiotherapist and non-specialized physiotherapists regarding several aspects, where specialists in general had higher interest and priority of scientific evidence, and perceived ability to interpret and evaluate scientific results.

The perspective on importance of a scientific approach, and views on to what extent clinical practice of physiotherapy in general in Sweden is based on scientific evidence were quite similar among specialists and non-specialists. Both specialists and non-specialists considered a scientific approach to be important within clinical practice, which is in line with previous Swedish studies [7, 18], but the majority considered that the general clinical practice in Sweden is based on scientific evidence only to a low or moderate extent (or not at all).

The scientific evidence was ranked as the highest prioritized foundation for clinical decision making among both specialists and non-specialists, although to a significantly higher extent among specialists. Also own clinical experience was ranked high as foundation. The clinical experience is related to the “clinical expertise” which is one of the three “legs” of EBM. The clinical expertise includes both the general basic skills of clinical practice and the individual experience and is considered to contribute to the balance and integration between different aspects to handle in the clinical decision-making [19]. The clinical expertise and experience may hence also be a valuable basis for the clinical application, especially if it is in line with existing evidence and/or no current evidence with sufficient quality exist. It is however important to be open for updates based on developed evidence, and to be aware that the individual clinical experience can be biased. The third leg; patient preferences, was ranked as a low priority for treatment selections. In a mixed method study regarding perspectives among Australian physiotherapists [20], participants considered evidence as important, but in their clinical decision making they also included patient expectations, colleagues’ treatment choices, and business demands. However, the patient expectations were also considered a major barrier for practical application of evidence in the clinic.

The general interest and perceived abilities to interpret and apply scientific information in clinical practice was higher among specialists than non-specialists. Physiotherapists with higher degree of education (Master and/or PhD) have, in previous studies, reported higher frequency of activities related to assimilation and application of scientific evidence [8] or higher evidence based practice dimension scores [21]. The specialist training includes at least a one-year master’s degree, which may contribute to strengthened scientific perspectives and critical thinking. In this previous study, physiotherapists with higher degree of education also rated their knowledge of EBM higher. However, even if the rated knowledge in general was high, a survey found that only 12–36% answered correctly regarding that EBM contains all the three components: clinical experience; use of the most reliable scientific evidence and patient’s preferences [8]. Rated or perceived knowledge may hence not be the same as actual knowledge. Earlier studies have also indicated that even though it is quite common to have confidence in appraising or interpreting scientific literature, a high proportions of clinicians also consider it difficult to interpret statistical results and/or or have low or limited research skills [4]. This also indicates a discrepancy between ability and practice.

To be able to assimilate and implement scientific results, both time and abilities are required. In our sample, the median number of scientific papers read during a regular month was one among non-specialists and three among specialists, which is approximately in line with previous Swedish studies [7, 8]. Especially among non-specialists, the perceived ability to interpret, evaluate and implement scientific results was not high among most participants. The perceived ability to interpret, evaluate and implicate scientific results was clearly higher among specialists. The cross-sectional design does not allow any causal conclusions, but the specialist education has likely contributed to the development of these skills. Based on the open comments, further education (including specialists training) also contributes to increased interest in science. However, it is also possible that this also works the other way around: clinicians with a higher interest in science continue with a specialist education. So, there can be a bi-directional causality which is not possible to further examine in a cross-sectional study.

The specialists in the sample had also a longer clinical experience, but this aspect did not seem to affect the differences between the groups. There are reasons to believe that specialists can serve as a knowledge bearer in the health care organizations and support less experienced colleagues in scientific matters, but only approximately 6% of Swedish registered physiotherapists hold a specialization and the majority (54%) of these are working in the Stockholm region [16, 17].

The ability to assimilate science is however needed, regardless of specialist level, since it is a foundation for evidence-based practice to be able to update knowledge of current scientific evidence. In previous studies, lacking knowledge and understanding of science has also been considered main barriers for implementation of EBM [9, 11–14]. Insufficient education within the topic has been lifted as one main inhibiting factor [11], which of course is related to the lacking knowledge and understanding. In our study, the views on whether the undergraduate program provided sufficient education in scientific methodology varied. Even though the program was considered to provide sufficient education, a continuous update and implementation is required to maintain and further develop the abilities through clinical practice [22]. An earlier study has pointed out the importance of promoting motivation for a scientific approach [6]. Some aspects may be required, or at least facilitate, to provide conditions for this development. Engagements from stakeholders to ingrain EBM in the clinical practice was lifted as an important aspect, from an expert view on the education of healthcare professionals. Integrating activities related to EBM in a structured way within everyday clinical practice can have the ability to promote a more consistent implication of the EBM [22]. A Swedish qualitative study that explored what supports physiotherapists use of research in clinical practice lifted aspects on both individual and workplace level [5]. On individual level, attitudes and motivation concerning research use and research-related knowledge and skills were considered supportive aspects. On workplace level, leadership support, organizational culture, research-related resources and knowledge exchange were lifted as supportive for the use of research in clinical practice. Among the physiotherapists in the interview study, available guidelines was found supportive [5]. A qualitative Danish study exploring barriers to use clinical guidelines for low back pain showed that a main perceived barrier was skepticism related to validity and applicability of the guidelines [23]. A critical view based on updated knowledge of scientific evidence is positive but requires knowledge and understanding of the science for a relevant skepticism. The ability to assess the quality of scientific evidence, and to critically appraise results and methodology is also defined as important foundation for the EBM [24].

Our participants rated lack of time as main barriers both for reading scientific papers and implementation of EBM to a higher degree. More allocated time was also rated as the main facilitating aspect for a scientific approach. This aspect has been lifted as main barriers for the implication of EBM within clinical practice in different professions [25]. However, time as such may not lead to increased EBM unless there are relevant competence; knowledge and understanding of the science. Also, more abilities to participate in further education, and access to scientific articles were considered as facilitating factors among almost half of the participants. Scientific conferences and general support and encouragement for EBM from the employer was considered facilitating among approximately one third. These are aspects that put the attention on how employers facilitate with a supporting work organization, that ensures and enables employees to be updated regarding EBM.

According to the “behavior change wheel” [26], changes in behavior are related to sources of behavior within the domains capability, opportunity, and motivation. This is much in line with the barriers and facilitators for applying science on clinical practice, that were reported in this study. The participants reported both individual and organizational aspects and this has also been reported in previous studies [5–10]. Interventions aiming to change behavior relate to several aspects including e g education, persuasion, incentivization, training, enablement, restrictions. A systematic meta-review examining barriers and facilitators to clinical behavior changes in primary care related to the theory of the behavior change wheel found that perceived barriers and facilitators were mainly related to the domains of capability and opportunity; factors related to knowledge, environmental context and resources, and social influences [27]. These aspects also relate to the perceived reasons for increased interest in science in our sample. A change in interest could be a step towards clinical behavior change and increased evidence based clinical practice.

In our sample, a better knowledge and understanding of scientific results were considered facilitating among a smaller proportion (less than one third), which may indicate that the majority already consider their knowledge sufficient. Although a lower proportion among non-specialists rated their ability to translate/implement results from scientific studies in clinical practice as high or very high, most of both non-specialists and specialists considered their knowledge and understanding sufficient for making decisions regarding clinical treatment based on scientific evidence. The interest and perceived importance of scientific approach was also mainly rated high. However, there were still a considerable share, especially among non-specialists, who considered their understanding of evaluation and interpretation of scientific results as low or moderate. The perceived knowledge was rated on approximately similar level as among Australian physiotherapists in a study from 2006 [28]. This competence is probably even lower in the general population of physiotherapists, since our sample most likely are affected by a non-response bias leading to over-representativity of physiotherapists with interest in science. Even though the majority of participants in our study considered their knowledge/understanding of research sufficient to make decisions regarding treatment based on a scientific basis, improved skills would probably make it easier to more effectively assimilate the scientific information, and also do it to a higher degree. Knowledge and understanding of the science are central conditions that often need further development not only during education but also during the clinical practice [22, 29]. In Sweden, physiotherapists in primary health care serve as first-line treatment, and no referrals are needed. Physiotherapies must therefore be updated on the most recent research in both diagnostics and treatment. Clinical decision making is not only a matter for the individual physiotherapists, and should be digested in a professional context where pros and cons for different treatment options are continuously discussed [30]. Shared decision making has become more and more recognized in a clinical context [31] and enables the patient to fully participate in his or her treatment. This highlights the importance of a solid EBM-basis, with knowledge and understanding of science as a fundament to be able to apply the scientific evidence. It also requires that the health care professional is updated regarding current state of science, to provide the patient with a solid decision basis to discuss.

For the majority of participants, the interest in science had increased during the years of clinical practice. The main reported reasons for increased interest were an aspiration to increase and update knowledge of scientific evidence for best clinical practice, and the clinical practice as such that had provided better insight and interest in need of knowledge and understanding about specific clinical topics. However, also further education such as courses, research involvement and specialist training were to large extent reported as reasons for increased interest in research. Hence, even if the higher interest and perceived ability and assimilation of research among specialists that we found may be because physiotherapists with higher interest for further education and research may be more encouraged to become specialists, the specialist training as such seem to contribute to the increased interest and understanding of science.

### Strength and limitations

Our study includes a relatively large sample, compared to other studies within this topic. However, the sample is most likely not representative for the total population of clinically active physiotherapists in Sweden. The response rate was low, and physiotherapists with a higher interest in science probably responded to a higher degree which may overestimate the positive approach. The descriptive results should hence not be directly generalized to the whole professional corps. Such non-response rate bias may be an issue in different forms of descriptive questionnaire surveys.

The main part of the respondents was recruited by the invitation from Physiotherapy Sweden, but only about two thirds of all registered physiotherapists in Sweden are members of this association. We used social media to also reach non-members, but these respondents were a minor part of the total sample. The complementary stratified analyses of responders via email invitation from The Swedish Association of Physiotherapists vs social media showed no clear differences (results not presented), which indicate that potential non-response bias did not differ mainly between the recruitment paths.

A majority of the respondents were women, which is in line with the gender distribution among all registered physiotherapists in Sweden. The proportion of specialist physiotherapists was however higher in the sample than in the total population of Swedish physiotherapists [17] which may also have affected the results for the total sample.

Lack of time, that is lifted as inhibiting factors both for reading scientific papers and a clinical practice based on scientific evidence to a higher extent, may also have contributed to a low response rate. However, the survey did not take long time to respond to. Despite a potential non-representative sample, the responses may still provide an insightful view of perspectives on the scientific approach. Also, the group comparisons (between specialists and non-specialists) within the sample are likely less affected by the non-response bias.

The questionnaire was created for this specific study, with the aim to focus more on the perspectives on science than the whole/general EBM process and is hence not tested for reliability or validity. Most part of the questionnaire was also developed with the aim to collect and present more direct opinions or perceptions of the defined item/topic, rather than any form of general/combined evaluation. As brought up in the discussion in a study examining adherence to clinical guidelines, forms of assessments of guideline adherence differ between studies, which may give different results [32]. Regarding perceived knowledge and understanding of science and application of EBM, the perceptions may differ from actual knowledge, practical skills or applications. As mentioned, previous studies have shown differences between perceived and actual knowledge [8]. However, the main aim with this study was to examine the perspective and attitudes, which we expect to be more related to the reported perceptions and answers.

## Conclusion

Among respondents, a scientific approach was considered valuable within physiotherapy but was not considered to be fully applied in clinical practice. The low response rate may however have resulted in a selection bias regarding interest in science and limits the generalizability of those results to the whole Swedish physiotherapist population. Lack of time was considered the most inhibiting factors for assimilation of scientific evidence. Specialist physiotherapists had in general significantly higher interest and priority of scientific evidence, and perceived ability to interpret and evaluate scientific results. Further education and specialist training after bachelor graduation hence seem to increase both interest in, and understanding of, science and may be a step towards clinical behavior change and strengthened conditions for evidence based clinical practice.

## Data Availability

Data is not publicly available, but if justified reason, corresponding author can be contacted to get access to anonymized data.

## Abbreviations

EBM: Evidence based medicine
IQR: Inter quartile range
Md: Median
SD: Standard deviation

## References

[1] Sackett DL, Rosenberg WM, Gray JA, Haynes RB, Richardson WS (1996). Evidence based medicine: what it is and what it isn't. BMJ.

[2] Djulbegovic B, Guyatt GH (2017). Progress in evidence-based medicine: a quarter century on. Lancet.

[3] Evidence-Based Medicine Working G (1992). Evidence-based medicine. a new approach to teaching the practice of medicine. JAMA.

[4] Condon C, McGrane N, Mockler D, Stokes E (2016). Ability of physiotherapists to undertake evidence-based practice steps: a scoping review. Physiotherapy.

[5] Dannapfel P, Peolsson A, Nilsen P (2013). What supports physiotherapists' use of research in clinical practice? a qualitative study in Sweden. Implement Sci.

[6] Dannapfel P, Peolsson A, Stahl C, Oberg B, Nilsen P (2014). Applying self-determination theory for improved understanding of physiotherapists' rationale for using research in clinical practice: a qualitative study in Sweden. Physiother Theory Pract.

[7] Bernhardsson S, Johansson K, Nilsen P, Oberg B, Larsson ME (2014). Determinants of guideline use in primary care physical therapy: a cross-sectional survey of attitudes, knowledge, and behavior. Phys Ther.

[8] Nilsagård Y, Lohse G (2010). Evidence-based physiotherapy: a survey of knowledge, behaviour, attitudes and prerequisites. Adv Physiother.

[9] da Silva TM, Costa Lda C, Garcia AN, Costa LO (2015). What do physical therapists think about evidence-based practice?. A systematic review Man Ther.

[10] Scurlock-Evans L, Upton P, Upton D (2014). Evidence-based practice in physiotherapy: a systematic review of barriers, enablers and interventions. Physiotherapy.

[11] Alshehri MA, Alalawi A, Alhasan H, Stokes E (2017). Physiotherapists' behaviour, attitudes, awareness, knowledge and barriers in relation to evidence-based practice implementation in Saudi Arabia: a cross-sectional study. Int J Evid Based Healthc.

[12] Diermayr G, Schachner H, Eidenberger M, Lohkamp M, Salbach NM (2015). Evidence-based practice in physical therapy in Austria: current state and factors associated with EBP engagement. J Eval Clin Pract.

[13] Ramirez-Velez R, Bagur-Calafat MC, Correa-Bautista JE, Girabent-Farres M (2015). Barriers against incorporating evidence-based practice in physical therapy in Colombia: current state and factors associated. BMC Med Educ.

[14] Silva TM, Costa LC, Costa LO (2015). Evidence-Based Practice: a survey regarding behavior, knowledge, skills, resources, opinions and perceived barriers of Brazilian physical therapists from Sao Paulo state. Braz J Phys Ther.

[15] Fysioterapeuterna. Physiotherapy education in Sweden https://www.fysioterapeuterna.se/In-English/Education: Swedish Association of Physiotherapists; 2022.

[16] Socialstyrelsen. Bedömning av tillgång och efterfrågan på legitimerad personal i hälso- och sjukvård samt tandvård -Nationella planeringsstödet 2021 (Assessment of supply and demand for licensed personnel in healthcare and dental care - National planning support 2021). 2021. Contract No.: 2021–2–7200.

[17] Fysioterapeuterna. Statistik om specialistutbildade fysioterapeuter från Fysioterapeuternas medlemssystem 28 januari 2022 (Statistics on specialist-trained physiotherapists from the Physioterapeuteren's member system 28 January 2022). 2022.

[18] Heiwe S, Kajermo KN, Tyni-Lenne R, Guidetti S, Samuelsson M, Andersson IL (2011). Evidence-based practice: attitudes, knowledge and behaviour among allied health care professionals. Int J Qual Health Care.

[19] Haynes RB, Devereaux PJ, Guyatt GH (2002). Clinical expertise in the era of evidence-based medicine and patient choice. Vox Sang.

[20] Gleadhill C, Bolsewicz K, Davidson SRE, Kamper SJ, Tutty A, Robson E (2022). Physiotherapists' opinions, barriers, and enablers to providing evidence-based care: a mixed-methods study. BMC Health Serv Res.

[21] Fernandez-Dominguez JC, De Pedro-Gomez JE, Jimenez-Lopez R, Romero-Franco N, Bays Moneo AB, Oliva-Pascual-Vaca A (2022). Physiotherapists' evidence-based practice profiles by HS-EBP questionnaire in Spain: a cross-sectional normative study. PLoS ONE.

[22] Lehane E, Leahy-Warren P, O'Riordan C, Savage E, Drennan J, O'Tuathaigh C (2019). Evidence-based practice education for healthcare professions: an expert view. BMJ Evid Based Med.

[23] Hubeishy MH, Rolving N, Poulsen AG, Jensen TS, Rossen CB. Barriers to the use of clinical practice guidelines: a qualitative study of Danish physiotherapists and chiropractors. Disabil Rehabil. 2022:1–10. 10.1080/09638288.2022.2157501 36537245

[24] Nunan D, O'Sullivan J, Heneghan C, Pluddemann A, Aronson J, Mahtani K (2017). Ten essential papers for the practice of evidence-based medicine. Evid Based Med.

[25] Engels C, Boutin E, Boussely F, Bourgeon-Ghittori I, Couturier B, Fromantin I (2020). Use of evidence-based practice among healthcare professionals after the implementation of a new competency-based curriculum. Worldviews Evid Based Nurs.

[26] Michie S, van Stralen MM, West R (2011). The behaviour change wheel: a new method for characterising and designing behaviour change interventions. Implement Sci.

[27] Mather M, Pettigrew LM, Navaratnam S (2022). Barriers and facilitators to clinical behaviour change by primary care practitioners: a theory-informed systematic review of reviews using the theoretical domains framework and behaviour change wheel. Syst Rev.

[28] Iles R, Davidson M (2006). Evidence based practice: a survey of physiotherapists' current practice. Physiother Res Int.

[29] Thomas A, Saroyan A, Dauphinee WD (2011). Evidence-based practice: a review of theoretical assumptions and effectiveness of teaching and assessment interventions in health professions. Adv Health Sci Educ Theory Pract.

[30] Thompson J, Yoward S, Dawson P (2017). The Role of physiotherapy extended scope practitioners in musculoskeletal care with focus on decision making and clinical outcomes: a systematic review of quantitative and qualitative research. Musculoskeletal Care.

[31] Hoffmann T, Bakhit M, Michaleff Z (2022). Shared decision making and physical therapy: What, when, how, and why?. Braz J Phys Ther.

[32] Husted M, Rossen CB, Jensen TS, Mikkelsen LR, Rolving N (2020). Adherence to key domains in low back pain guidelines: A cross-sectional study of Danish physiotherapists. Physiother Res Int.

